# Low Reproductivity of Giant Pandas May Be Associated with Increased Vaginal *Escherichia-Shigella*

**DOI:** 10.3390/microorganisms12122500

**Published:** 2024-12-04

**Authors:** Wei Wu, Fei Xue, Chong Huang, Yanshan Zhou, Guanwei Lan, Wenlei Bi, Jiabin Liu, Xiang Yu, Zusheng Li, Long Zhang, Feifei Feng, Jiang Gu, Rui Ma, Dunwu Qi

**Affiliations:** Sichuan Key Laboratory of Conservation Biology for Endangered Wildlife, Chengdu Research Base of Giant Panda Breeding, Chengdu 610081, China; happyeffiewu@163.com (W.W.); xf19880825@gmail.com (F.X.); chong_huang718@163.com (C.H.); zhouyanshan_gsau@163.com (Y.Z.); gw.lan@foxmail.com (G.L.); biwenlei@sina.cn (W.B.); jiabin_liu2013@126.com (J.L.); yuxiang9212@163.com (X.Y.); daisheng3@gmail.com (Z.L.); longzhang_csuft@163.com (L.Z.); fengfeifei2018@163.com (F.F.); gujiang1205@yeah.net (J.G.)

**Keywords:** giant panda, vaginal microbiome, pregnancy outcome, captive wildlife, ex situ conservation

## Abstract

The poor reproductive capacity of giant pandas significantly hinders the development of captive populations, with 80.88% of adult individuals being unable to successfully become pregnant and deliver offspring. The disturbance of vaginal microbiota has been proven to potentially lead to miscarriage, abortion, and stillbirth in mammals. To elucidate the potential relationship between the vaginal microbiota and the reproductive capacity of giant pandas, we performed high-throughput sequencing of vaginal microbiota at the time of fertilization and conducted comparative analyses based on different pregnancy outcomes. We found that the microbial diversity in the delivery (D) group exceeded that in the non-delivery (ND) group and the vaginal microbial community structure was statistically different between the two groups. The vaginal microbiota in the delivery pandas consisted of *unclassified Pseudomonadaceae* which was gradually replaced by the *Escherichia-Shigella* type of vaginal microbiota in the ND group. A function predictions analysis showed that infectious disease, glycan biosynthesis, and metabolism were significantly enriched in the ND group. Additionally, an analysis of the microbial community phenotypic categories indicated that the ND group exhibited a significantly higher abundance of Gram-negative bacteria, facultative anaerobes, potential pathogens, and stress-tolerant species compared to the D group, predominantly driven by the elevated abundance of *Escherichia-Shigella*. *Escherichia-Shigella* can be used within LDA and ROC analyses to diagnostically distinguish the vaginal microflora associated with bad pregnancy outcomes during estrus. Our results will help to identify potential pathogens causing reproductive tract diseases, reduce the number of reproductive tract disease infections in pandas, and increase the birth rate of giant pandas in conservation breeding programs.

## 1. Introduction

The population of captive giant pandas (*Ailuropoda melanoleuca*) has experienced steady growth over the past 30 years, benefiting from the successful implementation of ex situ conservation efforts and advancements in artificial breeding techniques [1,2]. However, approximately 81% of captive female pandas are unable to become pregnant or to give birth [3,4]. In previous research, the low fertility of female pandas has often been related to the content of different concentrates, range of activity, cortisone content, and the quality of male pandas’ sperm [5]. So far, the comprehensive diversity and structure of the vaginal microbiota within adult female giant pandas with different pregnancy outcomes have yet to be reported.

It is known that an animal’s vaginal microbiota helps to fend off pathogens and execute important physiological functions for the host, including functions associated with immunity, metabolism, and epithelial development [6]. Studies have shown that disorders affecting the vaginal bacterial community may be related to reproductive tract infections and affect pregnancy outcomes [7,8]. Alternatively, an abnormal vaginal microbiota may negatively affect the clinical pregnancy rate of in vitro fertilization patients [9]. Patients with bacterial vaginosis have been shown to have a greater microbiotic diversity and there are significant positive associations between certain microbial clusters and preterm birth [10]. Under certain conditions, some opportunistic bacteria, such as *Escherichia-Shigella* present in the vagina can be transformed into a pathogen, thereby impacting reproductive health [11]. Female infertility may be caused by *Escherichia-Shigella* through several mechanisms, including the disruption of the vaginal microbiota balance, interference with sperm function, and an increased risk of infections, and may have potential effects on endocrine function [11,12,13,14].

Compared with human vaginal bacterial communities, the vaginal microbes in livestock, companion animals, and wild animals are significantly different [15,16,17]. Drawing on the important role of vaginal *Lactobacillus* in human females, previous studies have isolated and cultured vaginal *Lactobacillus* from giant pandas. It was found that the vaginal *Lactobacillus* of giant pandas are primarily composed of *Lactobacillus salivarius GPV01*, *Lactobacillus plantarum GPV02*, *Lactobacillus saerimneri GPV03*, *Lactobacillus acidophilus WAV01*, *Lactobacillus crispatus WAV02*, and *Lactobacillus plantarum WAV03* [18]. However, subsequent studies revealed that the lactobacilli in the panda’s vagina were not the dominant species in terms of their relative abundance [19]. Our preliminary research indicated that the abundance and diversity of the vaginal microflora of giant pandas in estrus were significantly lower than those in non-estrus pandas, and the abundance of *Escherichia-Shigella* in the vagina remained low in non-estrus pandas, but increased significantly in estrus pandas [20].

At present, the relationship between the composition of vaginal microbiota during fertilization in giant pandas and pregnancy outcomes remains unclear. It is also unknown whether an increase in *Escherichia-Shigella* is associated with adverse pregnancy outcomes. To verify the above hypothesis, we used 16S rRNA sequencing to comprehensively characterize the bacterial composition, abundance, and structure of the vaginal microbiota in estrus female giant pandas with different pregnancy outcomes. This approach aimed to identify bacterial shifts associated with delivery and non-delivery. Our findings expand our understanding of the vaginal bacterial ecology in estrus female giant pandas and provide insights for improving the chances of successful delivery.

## 2. Materials and Methods

### 2.1. Animal Management

All of the individuals in this study were housed in adjacent traditional enclosures, which contained an indoor space with a wooden bed and an outdoor yard with grass, trees, shrubs, herbs, 2 wooden platform for feeding, several wooden climbing apparatuses, and a small cement water pond. All subjects were provided with a daily diet consisting of two steamed buns, two apples, and ad libitum bamboo. As ascertained by the veterinarian, every individual was in good health and had been free from antibiotics for a period of three months before the commencement of the study. None of the methods employed in this study or those that were significantly modified had any impact on the behaviors or management of the subjects involved. The sample collection procedures received approval from the Institutional Animal Care and Use Committee of the Chengdu Research Base of Giant Panda Breeding on 22 September 2018 (with the IACUC No. 201806). Throughout the sampling and experimental procedures, none of the pandas were subjected to any harm.

### 2.2. Sampling

Samples for vaginal microbial analyses were collected from 12 adult female giant pandas (age range from 6 to 17) housed at the Chengdu Research Base of Giant Panda Breeding, Chengdu, China. Each selected individual demonstrated special behaviors that indicated the onset of estrus, such as irritability, loss of appetite, howling, and frequent use of odor markers. Sample collection was performed after anesthesia. None of the pandas had records of antibiotic administration in the 3 months before sampling. Bacterial samples from the vagina were collected within 5 cm of the vaginal vulva using a disposable sterile swab. The samples were immediately transferred to the laboratory on dry ice and stored in a −80 °C refrigerator prior to the next step. The study individuals were divided into a delivery group (D, *n* = 6) and a non-delivery group (ND, *n* = 6) according to pregnancy outcome.

### 2.3. DNA Extraction and 16S rRNA Gene Sequencing

DNA was extracted from the swabs following the procedure detailed by Verdon et al. [21] with the employment of the Tiangen TIANamp Swab DNA Kit (manufactured by Tiangen, Beijing, China) and in strict accordance with the kit’s protocols for DNA isolation. The quality of DNA in each sample was evaluated through 1% agarose gel electrophoresis. Subsequently, the samples were dispatched to Shanghai Majorbio Bio-pharm Technology Co., Ltd. (located in Shanghai, China) for further tests for concentration, assessment, amplification, and sequencing, all of which were carried out according to their standard procedures [22,23]. The V3–V4 regions of the 16S rRNA gene were amplified using the primers 338F (5′-ACTCCTACGGGAGGCAGCAG-3′) and 806R (5′-GGACTACGCGGGTATCTAAT-3′), which are designed to target the conserved sequences present in bacteria. PCR reactions were conducted in triplicate 20 μL mixtures, each containing 4 μL of 5× FastPfu Buffer, 2 μL of 2.5 mM dNTPs, 0.8 μL of each primer (with a concentration of 5 μM), 0.4 μL of FastPfu Polymerase, and 10 ng of template DNA. Afterwards, the amplicons were retrieved from 2% agarose gels and further purified using the AxyPrep DNA Gel Extraction Kit (produced by Axygen Biosciences, Union City, CA, USA). Their quantification was performed using QuantiFluor-ST (manufactured by Promega, Madison, WI, USA) following the relevant protocols. Purified amplicons were combined in equimolar amounts and subjected to paired-end sequencing (2 × 300) on an Illumina MiSeq platform (manufactured by Illumina, San Diego, CA, USA) as per the instructions. The raw reads were then deposited into the NCBI Sequence Read Archive (SRA) database (with the Accession Number: SUB8319744).

### 2.4. Statistical Analysis

Raw reads were demultiplexed and quality-filtered via QIIME (version 1.9.1). The taxonomy of each 16S rRNA gene sequence was analyzed against the SILVA 128/16s bacteria database, with a confidence threshold of 70%. After setting the smallest sequencing depth and clustering, all operational taxonomic units (OTUs) at 97% identity were obtained using UPARSE (version 7.0). The shared and unique OTUs between groups were visualized with Mothur software (version 1.31.2). R software (v3.6.1) was employed to draw a ranked abundance curve to explain species richness and evenness. Alpha diversity and richness were calculated by means of Wilcoxon rank-sum test in the said software. UPGMA cluster analysis with the Bray–Curtis distance matrix was executed in R (v3.6.1). In R (v3.6.1), beta diversity was compared with the representative sequences of OTUs for each group through the Vegan package, and the difference between the two groups was investigated using the Analysis of Similarities (ANOSIM) and Nonparametric Multivariate Analysis of Variance (Adonis). After 999 iterations, Non-metric Multidimensional Scaling Analysis (NMDS) display diagrams were obtained to study the similarity or dissimilarity of sample community composition among samples. To explore the differences between the two groups regarding the bacteria with the top 5 relative abundance on a phylum level and the top 10 relative abundance on a genus level, Wilcoxon rank-sum tests were carried out in R (v3.6.1). LEfSe software 1 (version 1.0) was used to make an LEfSe linear discriminant analysis (LDA) diagram. The analysis of the Receiver Operating Characteristic Curve (ROC) model was performed with Graphpad Prism 9. Microbial functions were predicted by PICRUSt (version 1.0.0) and matched to the Kyoto Encyclopedia of Genes and Genomes (KEGGs) database. The functional differences between groups were ascertained by Linear Discriminant Analysis (LDA) on Galaxy Online (version 1.0.0). The phenotypes of the organism-level microbiome were predicted and compared with Bugbase [24]. Comparisons were made between the proportions of six phenotypic categories—Gram staining, oxygen tolerance, biofilm formation ability, mobile element content, pathogenicity, and oxidative stress tolerance—among the delivery and non-delivery groups. In this study, differences were deemed significant when *p* < 0.05 and extremely significant when *p* < 0.01.

## 3. Results

### 3.1. OTU Characteristics of D and ND Groups

**Figure 1:**
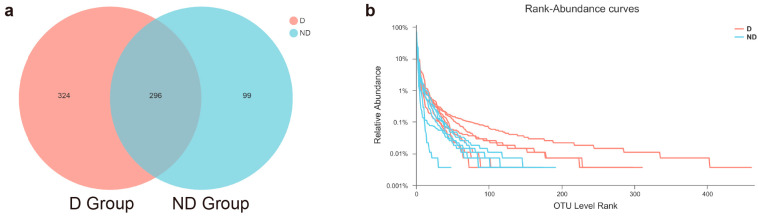
(**a**) OTU distribution in the two groups. The red and light blue circles represent samples of vaginal swabs in the D and ND groups, respectively. The overlap indicates the OTUs shared by the two groups. (**b**) Rank–abundance curve. Curves of different colors display the relative percentage of the number of species (Y-axis) for each sample at the rank of the number of species (X-axis). The position of the horizontal coordinate at the end of the sample curve’s extension is the number of species in the sample. A smoother decline of the curve implies higher species diversity of the sample, whereas a rapid and steep decline indicates a high proportion of dominant colonies in the sample and a lower diversity.

After filtering and splicing the raw data, 517,354 high-quality tags were produced for both the D and ND groups. The average number of sequences per sample was 43,113, varying from 32,167 to 54,453 (Table S1). The average sequence length was 425 bp, with the longest being 490 bp and the shortest being 207 bp. In total, 719 OTUs were obtained, 296 of which were shared by the two groups, and 324 and 99 OTUs were uniquely identified in the D and ND groups, respectively (Figure 1a).

### 3.2. Alpha Diversity Differences in the D and ND Groups

The Sobs, Chao, and ACE richness indices for the D group were higher than those for the ND group (Table 1), yet no significant difference was found between the groups in the Wilcoxon test. However, significant differences existed between the D and ND groups in the Shannon index (2.59 vs. 1.89, *p* = 0.03) and the Simpson index (0.18 vs. 0.30, *p* = 0.04), demonstrating a higher bacterial diversity in the delivery group’s vaginal samples compared to the non-delivery samples. The Good’s coverage estimator for each group exceeded 99%, suggesting that the current sequencing depth was sufficient to capture the bacterial diversity of the vaginal swabs. Additionally, the Simpson index indicated that the bacterial community distribution in the samples was highly uneven, as also seen in the rank–abundance curve with a high slope and a long tail of low-abundance OTUs (Figure 1b). The 100 most abundant OTUs accounted for 96.99% of all sequences, and most of the remaining OTUs were of low abundance.

### 3.3. Community-Composition Differences in D and ND Group

**Figure 2:**
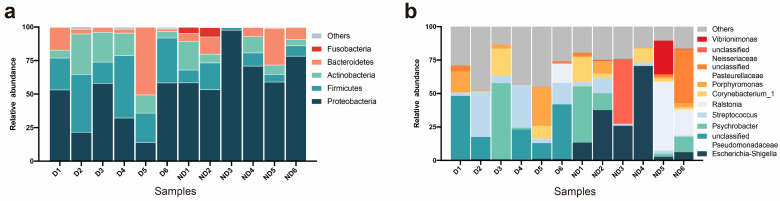
Microbial community bar plots. (**a**) Phylum-level microbial community bar plot. Bar charts depict the relative abundances of all detected phyla in the vaginas collected from estrus female giant pandas in the D (D1–D6) and ND (ND1–ND6) groups. The identities of the microbiome are indicated by the colored blocks on the right. (**b**) Genus-level microbial community bar plot. Bar charts present the relative abundances of all detected genera in the vaginas collected from the D (D1–D6) and ND (ND1–ND6) groups. The identities of the microbiome are shown with the colored blocks on the right.

**Figure 3:**
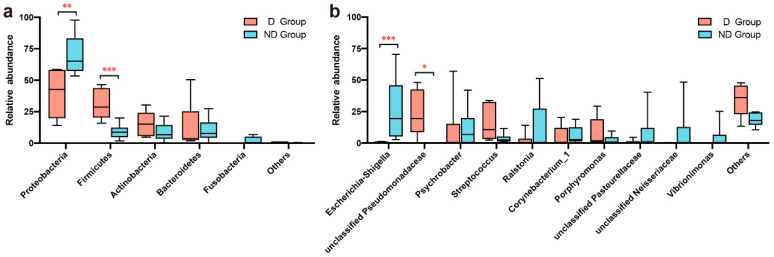
(**a**) Comparison of bacterial differences among different groups of samples at the phylum level among the 5 most abundant genera. (**b**) Comparative analysis of different species of bacteria among groups at the genus level among the top 10 most abundant genera (0.01 < *p* < = 0.05, marked with “*”; 0.001 < *p* < = 0.01, marked with “**”; *p* < 0.001, marked with “***”; if *p* > 0.05, not marked).

A characterization of the bacterial distribution was carried out based on relative taxonomic abundances. A total of 23 phyla, 41 classes, 86 orders, 161 families, 368 genera, 534 species, and 719 OTUs were identified in all vaginal samples. At the phylum level of comparison, the D and ND groups shared the same top four bacterial species, albeit with differences in their overall relative abundance. In the D group, Proteobacteria (39.71%) were the most dominant phyla in the samples, followed by Firmicutes (30.75%), Actinobacteria (15.60%), Bacteroidetes (13.23%), and Cyanobacteria (1.10%; Figure 2a). In the ND samples, Proteobacteria (69.74%) were also the most abundant phyla in terms of their relative abundance, followed by Bacteroidetes (10.32%), Firmicutes (9.13%), Actinobacteria (8.64%), and Fusobacteria (1.95%; Figure 2a). The estimated cumulative abundance of these dominant phyla was over 99% of the identified OTUs. The vaginal bacterial communities of giant pandas in estrus for the D and ND groups were similar in composition, but there was a difference in their relative abundances at the phylum level. A further analysis of the relative abundance showed that the relative abundance of Proteobacteria in the ND group was significantly higher than that in the D group (*p* = 0.013); on the other hand, the relative abundance of Firmicutes in the D group was significantly higher than that in the ND group (*p* = 0.002; Figure 3a).

At the genus level, a total of 368 bacterial genera were identified, 322 in the D group and 243 in the ND group. The vaginal samples from the D group had the highest *unclassified Pseudomonadaceae* (23.30%) content, followed by *Streptococcus* (15.53%), *Psychrobacter* (9.76%), *Porphyromonas* (8.20%), and *Corynebacterium_1* (5.08%). In comparison, the D group had the highest amount of *Escherichia Shigella*, accounting for 25.99% of the total microbial relative abundance, the second most relative abundant was *Ralstonia* (11.80%), followed by *Psychrobacter* (11.38%), *unclassified Neisseriaceae* (8.23%), and *unclassified Pasteurellaceae* (7.49%; Figure 2b). Based on the analysis of relative abundance at the genus level, the top 10 species in all samples were chosen to assess the significance of the difference test between the two groups. The relative abundance of *Escherichia-Shigella* in the ND group was significantly higher than that in the D group (*p* = 0.005; Figure 3b), while the relative abundance of *unclassified Pseudomonadaceae* in the D group was significantly higher than that in the ND group (*p* = 0.045; Figure 3b). Our results indicated that the vaginal bacterial community in the delivery group differs from that in the non-delivery group at the genus level.

### 3.4. Bacterial Community Structure Differences in the D and ND Groups

**Figure 4:**
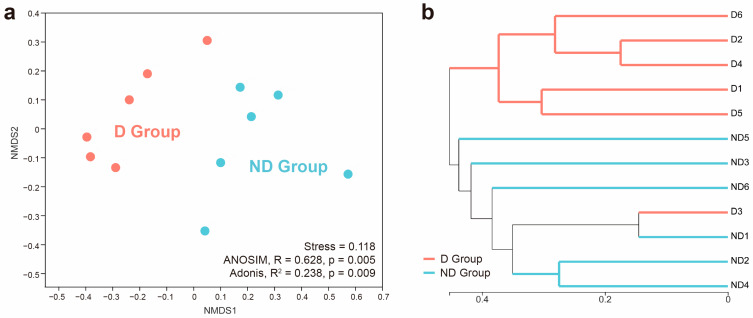
(**a**) Non-metric multidimensional scaling analysis (NMDS) based on the Bray–Curtis distance matrix. (description: X-axis is NMDS1 and Y-axis is NMDS2). The scales of the X-axis and Y-axis are, respectively, the projection coordinates of sample points in the two-dimensional plane. Each dot represents a sample, with different colors denoting different groups. (**b**) Sample clustering results (description: Bray–Curtis). The same color indicates samples in the same group. A short distance between samples implies a high similarity.

No overlap was found among the microbiota of the samples, and a clear segregation in community structures was exhibited between the two groups (Figure 4a). A significant separation occurred between the groups in the ANOSIM (R = 0.628, *p* = 0.005) and Adonis (R^2^ = 0.238, *p* = 0.009) testing. The results for the UPGMA cluster number also demonstrated that the majority of samples in the same group possess similar branches (Figure 4b). Furthermore, there was an obvious clustering pattern in the D group’s vaginal bacterial community, which was the same as that in the ND group.

### 3.5. Potential Biomarkers of D and ND Group

**Figure 5:**
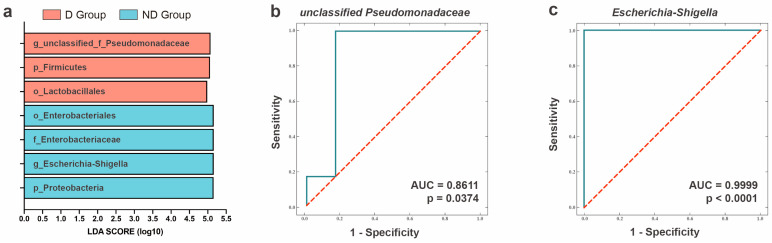
Identification of potential biomarkers using the LEfSe test and ROC. (**a**) Linear discriminant analysis (LDA) demonstrated distinct microorganism enriched in the D group and the ND group. The graph shows the LDA scores obtained from a linear regression analysis of the significant microorganism groups in the two groups. The initial letter or prefix of the bacterial names in the figure represents the current taxonomic level: p stands for phyla, c for classes, o for orders, f for families, and g for genera. When the default LDA value is more than 4.0 and the *p* < 0.05, the result corresponds to a differential species. Receiver operating characteristic curve (ROC) for comparing the accuracy of two potential biomarkers: (**b**) *unclassified Pseudomonadaceae* and (**c**) *Escherichia-Shigella*. (Description) The X-axis shows specificity and the Y axis shows sensitivity. The red dotted line represents the random selection probability; the blue line represents the model prediction probability; the area under the curve (AUC) represents the distinction between the different categories.

To identify potential biomarkers, an LDA and ROC model was created. The LDA results (Figure 5a) showed that the genera were significantly different in the vaginal samples from estrus female giant pandas with the delivery outcome (in the D group), which consisted of *unclassified Pseudomonadaceae*. Moreover, the results showed that there were significant difference in the vaginal samples from estrus female giant panda who did not deliver (in the ND group), which consisted of *Escherichia-shigella*. Two genera, *unclassified Pseudomonadaceae* and *Escherichia-shigella*, screened in the LDA, were used to construct a diagnostic model for differentiating the vaginal microbiota during estrus with delivery and non-delivery outcomes, respectively. The accuracy of the model was evaluated using ROCs. The results showed that the accuracy of the *Escherichia-Shigella* model (AUC = 0.9999) was higher than that of the *unclassified Pseudomonadaceae* model (AUC: 0.8611). These two genera can be used to diagnostically distinguish the vaginal microflora associated with different pregnancy outcomes during estrus.

### 3.6. Functional Capacity Differences in the D and ND Groups

**Figure 6:**
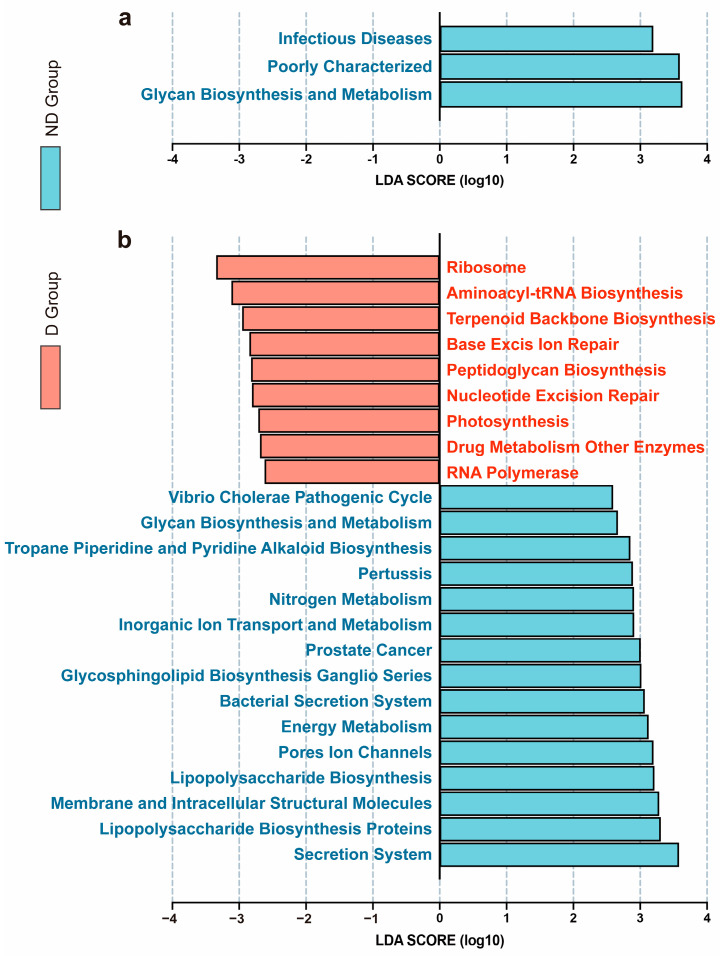
The changes in the function of vaginal microbiota between D and ND groups. LEfSe identified the pathways on (**a**) KEGG level 2 and (**b**) KEGG level 3 with the significant differences in abundance between the D and ND groups. (Red indicates the D group; light blue indicates the ND group; the predetermined threshold on the logarithmic linear discriminant analysis (LDA) score for discriminative features was set at >2.0; the predetermined *p* value was set at <0.05).

To predict the bacterial functions of the members of the different vaginal communities, a PICRUSt analysis was performed based on the 16S rRNA composition data for each sample. Among all the pathways on KEGG level 2, infectious disease, poor characterization, and glycan biosynthesis and metabolism were significantly enriched in the ND group (Figure 6a). Among all the pathways on KEGG level 3, nine pathways were significantly enriched in the D group including the ribosome, aminoacyl-tRNA biosynthesis, terpenoid backbone biosynthesis, base excision repair, peptidoglycan biosynthesis, nucleotide excision repair, photosynthesis, drug metabolism by other enzymes, and RNA polymerase (Figure 6b). Fifteen pathways were significantly enriched in the ND group including the vibrio cholerae pathogenic cycle, glycan biosynthesis and metabolism, tropane piperidine and pyridine alkaloid biosynthesis, pertussis, inorganic ion transport and metabolism, prostate cancer, glycosphingolipid biosynthesis ganglio series, the bacterial secretion system, energy metabolism, ion pore channels, lipopolysaccharide biosynthesis, membrane and intracellular structural molecules, lipopolysaccharide biosynthesis proteins, and the secretion system.

**Figure 7:**
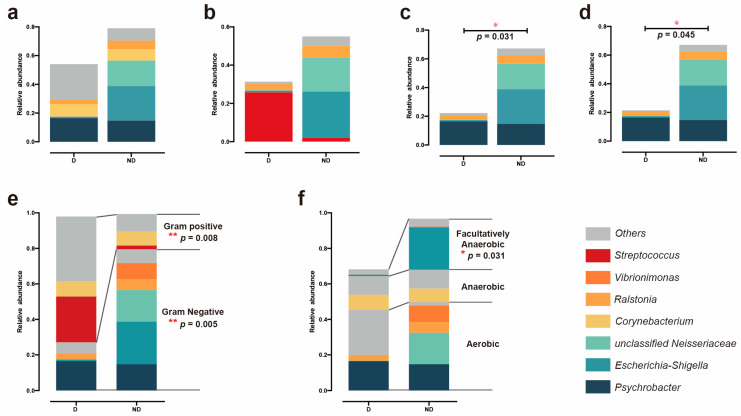
BugBase predictions of microbial community phenotypes and the corresponding bacterial contributions. (**a**) Biofilm formation. (**b**) Mobile genetic element content. (**c**) Pathogenic risk. (**d**) Oxidative stress tolerance. (**e**) Gram bacterial classification. (**f**) Oxygen utilization. (Only the top 10 genera in terms of relative abundance are shown with the color blocks on the right, the rest of the genera were described as others; the black lines in panels (**e**,**f**) represent the differences in the relative abundance of the corresponding bacterial genera between the D and ND groups; statistical significance was determined by Wilcoxon rank–sum test; 0.01 < *p* <= 0.05, marked with “*”; 0.001 < *p* <= 0.01, marked with “**”; if *p* > 0.05, not marked).

On the organism level, gene functions related to facultative anaerobiosis, being potentially pathogenic, and stress tolerance were depleted in the D group’s samples, chiefly due to reductions in reduced taxa within the genus *Escherichia-Shigella* and *unclassified Neisseriaceae* (Figure 7c,d,f). Gram-negative species were highly significantly enriched in the ND group’s samples, probably because of the increased abundances of *Escherichia-Shigella* and *unclassified Neisseriaceae* (Figure 7e). Moreover, significant decreases in Gram-positive species were predicted for the samples in the ND group, mainly due to the reduced abundance of *Streptococcus* (Figure 7e).

## 4. Discussion

The widespread application of 16S rRNA gene high-throughput sequencing has revealed many previously undiscovered bacterial groups, which will help to improve our understanding of the microbial communities hosted by endangered wildlife and present potential strategies to aid their conservation programs. In this study, 16S rRNA gene sequencing technology was used to analyze the vaginal bacterial diversity of female giant pandas in estrus to identify their relationships with pregnancy outcomes. The results of this study will inform future giant panda breeding efforts and provide a broader understanding of their reproductive system diseases.

As microorganisms are reliant on reproductive tract mucosal systems, uterine microbial community imbalances will affect vaginal microbial composition. For example, the vaginal microbial composition of sows with endometritis is different from healthy sows [25]. Vaginal microbial imbalances can also cause pathological changes in the uterus and even lead to negative pregnancy outcomes [7,8,26,27]. In this study, the vaginal microbiota of 12 female pandas in estrus were analyzed. With a guaranteed sequencing coverage of the two groups, the Alpha diversity analysis showed that the D group had a higher diversity (*p* < 0.05) and richness (*p* > 0.05) than the ND group, which agreed with the results reported for humans and other animals [28,29]. Notably, in this study, the Sobs, Ace, and Chao indices for the ND group did not show a significant decrease compared to the D group, whereas the Shannon and Simpson indices showed a significant decline. This result may suggest that although the evenness of species did not change significantly, the richness and community structure did [30]. Such changes could be due to shifts in the dominance of certain species within the vaginal microbiota of the ND group or adjustments in the relative abundance of species within the community [31]. These changes might be related to environmental factors, disease states, or other biological factors, such as progesterone [30,32,33]. Higher progesterone concentrations will increase the diversity and populations of microbes in the vagina, and the reduced bacterial diversity in the ND group may be due to insufficient individual progesterone secretion [34].

In this study, 23 bacterial phyla were represented in all vaginal samples. The four most predominant phyla were Proteobacteria, Firmicutes, Actinobacteria, and Bacteroidetes, constituting 99% of the total microbiota. Our findings are consistent with previous studies of eight estrus female pandas [19,23]. Compared to the vaginal microbial compositions of other animals, the panda’s is relatively special because of the presence of Actinobacteria and the high relative abundance of Proteobacteria [6,16,25,35,36].

At the genus level, the microflora of each sample is different but maintains some similarity. It can be seen from the sample cluster analysis that the genus compositions of the delivery samples are similar to each other. In both the Wilcoxon test and LDA, *Escherichia-Shigella* was enriched in the ND group. It has been reported that *Escherichia-Shigella* in the vaginas of giant pandas are significantly enriched during the estrus period [20]. *Escherichia-Shigella* is a common microorganism in the vaginas of many animals; however, an increase in the abundance of *Escherichia-Shigella* may cause disease [37,38]. There is a strong correlation between an increased *Escherichia-Shigella* abundance in vaginal microbes and the occurrence of endometritis in sows [25]. In agreement with the results of our study, *Escherichia-Shigella* is also considered to be a microbial marker that can be used as a diagnostic marker for missed miscarriage [39]. We speculate that the process of *Escherichia-Shigella* competing for dominance broke the original balance of the vaginal microbiota, causing the vaginal microbiota to be in a sub-healthy state. This may explain the enrichment of the infectious disease pathway on KEGG level 2 in the ND group. However, the sequencing results are only a snapshot of the change processes affecting the vaginal flora, the change in the composition of the microbiome in the non-delivery group did not reach a certain state, and it remained in the state of an “intermediate flora” [40,41,42]. Previous studies have clearly indicated that the presence of an abnormal vaginal microbiota is significantly associated with tubal factor infertility [9]. Bacteria from the intermediate microbiota may ascend to the upper reproductive tract, altering the microbial ecological environment of the fallopian tubes, and potentially contributing to infertility in females [9]. This may be the reason that the giant pandas in our study did not show any clinical symptoms of vaginal disease but did have different pregnancy outcomes.

In agreement with a previous report on giant pandas, we found the relative abundance of *Lactobacillus* in the vagina to be low and it did not contribute to the dominant genus [19]. In human female vaginal microorganisms, the activity of *Lactobacillus* is essential to protecting women from genital infections and to maintain the natural healthy balance of the vaginal flora by keeping the microenvironment at a low pH value [43,44]. Despite the low relative abundance of *Lactobacillus* in the giant panda vagina, other genera of the order *Lactobacillales,* such as *Streptococcus*, have a high relative abundance and belong to the dominant genus of vaginal bacteria. This resulted in a significant enrichment of *Lactobacillales* in the LDA results for the D group. *Streptococcus* was increased in the D group and performed a lactic acid-producing function, which is similar to the effect of *Lactobacillus* in humans [45]. This may illustrate that *Streptococcus* in the vagina of giant pandas may play an important role in their pregnancy outcomes.

Through a functional capacity analysis, Gram-negative species were found to be extremely significantly increased in the ND group, owing to the increased abundance of *Escherichia-Shigella* and *unclassified Neisseriaceae*. Meanwhile, the changes in the relative abundance of bacteria resulted in potentially pathogenic and stress-tolerant groups. There are various potential reasons for these changes; for example, deficiencies in estrogen production may lead to a decrease in the relative abundance of *Lactobacillales* in the vagina [46,47]. Interventions with antibiotics may not be the best way to control vaginal microbiota and risk increasing bacterial resistance. Hormone and microbiota transplantation may be the best way to improve delivery in the future; however, this requires further study [48,49].

In conclusion, our results have demonstrated, for the first time, the different vaginal microbial communities of giant pandas in estrus relative to different pregnancy outcomes. Several significant differences related to the relative abundances of the bacterium were found that may likely affect pregnancy outcomes, potentially reducing the success of conservation breeding programs. The description of the microbiota of the vagina of giant pandas in estrus presented in this study should aid in future investigations of reproductive disorders and bacterial diseases diagnosis, and improve estrus management in captive giant pandas. Our prospective study included only preterm female giant pandas in estrus. However, this study was based on previous knowledge of the vaginal microbiome being different in estrus and non-estrus giant pandas. Although the sample size in our study was relatively small, it was sufficient to meet the requirements of scientific statistical analyses, ensuring that the results are both authentic and scientific. Future studies must plan to incorporate amniotic fluid and placental samples to obtain a complete overview of the source of the female pandas’ vaginal microbiome. Since female giant pandas frequently use their tails to wipe perianal gland markers during estrus, the microorganisms in the environment and their behavior may have a certain influence on the composition of their vaginal microbiota. Future studies must explore the relationship between the captive environment, behavior, and vaginal microbiota of female giant pandas, and search for the possibility of changes in the type of vaginal microbiota from various perspectives, to change the current situation of infertility in captive populations.

## 5. Conclusions

The difficulty of achieving successful pregnancies in captive female giant pandas has been a significant barrier to the development of their population. Utilizing high-throughput sequencing of the 16S rRNA gene, this study is the first to reveal the association between the vaginal microbiota of estrous giant pandas and pregnancy outcomes. The vaginal microbial diversity in the delivery group (D group) was higher than that in the non-delivery group (ND group), with statistically significant differences in community structure. *Escherichia-Shigella* was enriched in the ND group and could serve as a biomarker for distinguishing vaginal microbiota associated with different pregnancy outcomes. The ND group exhibited higher abundances of Gram-negative bacteria, facultative anaerobes, potential pathogens, and stress-tolerant species, primarily driven by Escherichia-Shigella. The findings of this study contribute to identifying potential pathogens of reproductive tract diseases in giant pandas and are of great significance for improving the birth rate of captive giant pandas and advancing conservation breeding programs.

## Figures and Tables

**Table 1:** Alpha diversity indices for female estrus giant panda vaginal bacteria in each group at 97% identity by Wilcoxon rank–sum test.

Group	Sobs		ACE		Chao		Shannon		Simpson		Coverage	
	Mean	SD	Mean	SD	Mean	SD	Mean	SD	Mean	SD	Mean	SD
D	240.33	141.33	308.22	140.04	290.26	141.19	2.59	0.53	0.18	0.09	0.99	0
ND	135.00	51.19	202.88	88.20	180.44	70.85	1.89	0.50	0.30	0.12	0.99	0
*p*-value	0.30		0.17		0.23		0.03		0.04		0.3	

## Data Availability

The data presented in this study are openly available in the NCBI Sequence Read Archive (SRA) database at https://www.ncbi.nlm.nih.gov/bioproject/PRJNA855853 (Accession Number: SUB8319744).

## Supplementary Materials

The following supporting information can be downloaded at: https://www.mdpi.com/article/10.3390/microorganisms12122500/s1, Table S1. The 16S rDNA sequencing data of vaginal samples from delivery (D) and non-delivery (ND).
