# Supplementary material for: Low Reproductivity of Giant Pandas May Be Associated with Increased Vaginal Escherichia-Shigella
Source: Microorganisms. 2024 Dec 4;12(12):2500. doi: 10.3390/microorganisms12122500 (PMC11727807; doi:10.3390/microorganisms12122500)
Supplement: Supplementary file 1 [file microorganisms-12-02500-s001.zip › microorganisms-3279780-supplementary.pdf]

Low Reproductivity of Giant Pandas May Be Associated with Increased Vaginal *Escherichia-Shigella*

Authors

Wei Wu, Fei Xue, Chong Huang, Yanshan Zhou, Guanwei Lan, Wenlei Bi, Jiabin Liu, Xiang Yu, Zusheng Li, Long Zhang, Feifei Feng, Jiang Gu, Rui Ma*, Dunwu Qi*

Table S1 The 16S rDNA sequencing data of vaginal samples from delivery (D) and non-delivery (ND)

Sample ID	Sequence number	Base number	Mean length	Min length	Max length
D1	32167	13671887	425.028352	214	460
D2	37545	15828456	421.586256	337	479
D3	49696	21015641	422.883954	262	433
D4	33021	13995015	423.821659	334	450
D5	45852	19267799	420.217199	313	459
D6	33251	14189683	426.744549	253	437
ND1	43819	18559389	423.546612	225	431
ND2	49173	20890788	424.842658	219	470
ND3	45695	19596640	428.857424	207	452
ND4	49727	21174097	425.806845	239	490
ND5	54453	23189626	425.864984	402	435
ND6	42955	18322194	426.543918	219	441
